# A modified Gateway cloning strategy for overexpressing tagged proteins in plants

**DOI:** 10.1186/1746-4811-4-3

**Published:** 2008-01-22

**Authors:** Manu J Dubin, Chris Bowler, Giovanna Benvenuto

**Affiliations:** 1 Laboratory of Cell Signalling, Stazione Zoologica Anton Dohrn, Villa Comunale, Naples, Italy; 2 Department of Genetics, University of Kassel, Kassel, Germany; 3 CNRS UMR 8186, Molecular Plant Biology Laboratory, Ecole Normale Supérieure, Paris, France

## Abstract

**Background:**

Recent developments, including the sequencing of a number of plant genomes, have greatly increased the amount of data available to scientists and has enabled high throughput investigations where many genes are investigated simultaneously. To perform these studies, recombinational cloning methods such as the Gateway system have been adapted to plant transformation vectors to facilitate the creation of overexpression, tagging and silencing vectors on a large scale.

**Results:**

Here we present a hybrid cloning strategy which combines advantages of both recombinational and traditional cloning and which is particularly amenable to low-to-medium throughput investigations of protein function using techniques of molecular biochemistry and cell biology. The system consists of a series of twelve Gateway Entry cassettes into which a gene of interest can be inserted using traditional cloning methods to generate either N- or C-terminal fusions to epitope tags and fluorescent proteins. The resulting gene-tag fusions can then be recombined into Gateway-based Destination vectors, thus providing a wide choice of resistance marker, promoter and expression system. The advantage of this modified Gateway cloning strategy is that the entire open reading frame encoding the tagged protein of interest is contained within the Entry vectors so that after recombination no additional linker sequences are added between the tag and the protein that could interfere with protein function and expression. We demonstrate the utility of this system for both transient and stable Agrobacterium-mediated plant transformations.

**Conclusion:**

This modified Gateway cloning strategy is complementary to more conventional Gateway-based systems because it expands the choice of tags and higher orders of combinations, and permits more control over the linker sequence lying between a protein of interest and an epitope tag, which can be particularly important for studies of protein function.

## Background

The flood of information resulting from the sequencing of a number of plant genomes and other recent developments have led to a huge increase in the number of studies carried out using plants transformed with DNA fragments to overexpress or modulate the expression of a gene of interest. While the process of *Agrobacterium tumefaciens*-mediated plant transformation has improved over the last 15 years or so [1], the binary vectors used in this method are large (typically 10 to 19 kb) and often contain only a few unique restriction sites that are useful for cloning. As cloning constructs into these vectors is often an awkward and time-consuming process, some investigators have shifted to the Gateway site-specific recombination (SSR) cloning system commercialized by Invitrogen (Carlsbad, CA). In this system the construct of interest is first cloned into a minimal Entry vector (pENTR) and is then transferred to a Destination vector (pDEST) by site-specific recombination. This is done using the Int and Xis recombination proteins that recognize the *att*L and *att*R sites in Entry and Destination vectors, respectively, and that recombine them to generate *att*B sites [2]. The gene of interest is inserted into the Entry vector either using SSR with BP clonase or by cloning into the multiple cloning site (MCS) contained between the *att*L1 and *att*L2 sites by restriction enzyme and ligase (REaL) cloning. The entry clone can then be easily transferred to several Destination vectors by simple SSR reactions.

Gateway binary vectors have proved to be particularly useful for high-throughput plant transformation studies [3-6] where traditional REaL cloning methods would have been prohibitively time consuming. However, the use of these vectors has a number of drawbacks. For example, the presence of the *att*B recombination site between the tag and the gene of interest in the recombined Destination vectors results in a long (at least 8 and sometimes up to 20 amino acids) and often highly charged sequence which has the potential to result in the synthesis of a non-functional or insoluble protein-tag fusion and may also affect the expression levels of the protein of interest [7].

Here we report an alternative Gateway-based strategy that has been developed to create N- or C-terminally tagged protein overexpression constructs that lack *att*B-derived linker amino acids between the protein of interest and the tag. The *att*B sequences are avoided because the tag is contained within the Entry cassette instead of being in the Destination vector, and so the entire open-reading-frame (ORF) encoding the tagged protein of interest is generated within the Entry vector. In this system REaL cloning is first used to generate the fusion of the gene of interest with the tag, and SSR cloning is then used to transfer the ORF into a plant binary Destination vector containing a promoter and resistance cassette of choice, without concerns about reading frame. A total of twelve modified pENTR vectors have been generated, comprising six different tags for both N- and C-terminal fusions, and we show that these constructs can be used to study proteins of interest following introduction of these vectors into *Arabidopsis *or following transient *Agrobacterium*-mediated transformation and co-transformation in *Nicotiana benthamiana *leaves [8].

## Results

**Figure 1:**
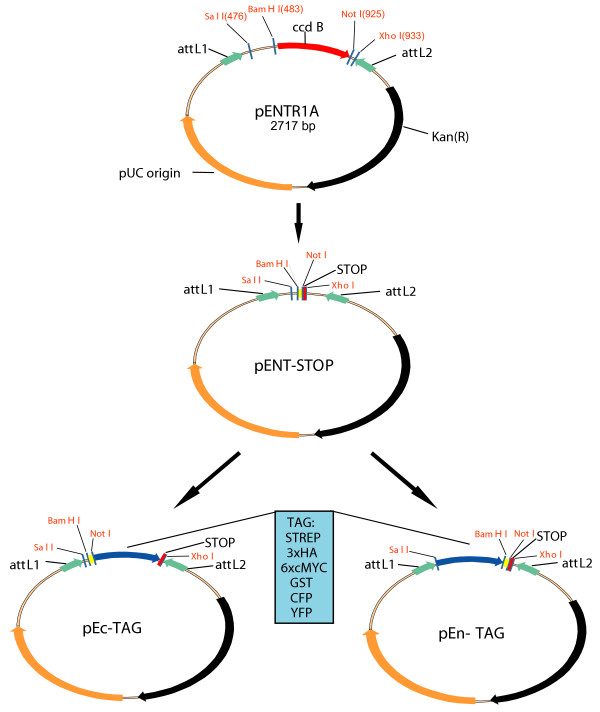
**Gateway Entry vector design**. Schematic drawing of the cloning strategy to generate modified Entry vectors. pENT-STOP vector was constructed starting from pENTR1A (Invitrogen) by introducing, in place of the *ccd*B toxicity gene, a short cloning site region and a STOP codon immediately after the *Not *I site. The six different tags were cloned in both the 3' orientation for C-terminal tagging (pEc-TAG) and 5' orientation for N-terminal tagging (pEn-TAG). All these vectors have the same cloning sites, *Bam*H I and *Not *I, to allow insertion of the gene of interest without stop codon both for N-terminal and C-terminal tagging because a stop codon is already present within the vectors.

In order to obtain modified Gateway Entry cassettes for epitope tagging, we first generated a pENT-Stop vector by introducing a short fragment into pENTR-1A (Invitrogen) in place of the *ccd*B toxicity gene (Figure 1) to generate a *Not *I site followed by a stop-codon. This vector has then been used as mother plasmid for subsequent cloning. Six different tags were introduced within this vector, as schematically illustrated in Figure 1, to allow a variety of experimental approaches. Enhanced Yellow Fluorescent Protein (EYFP) and Enhanced Cyan Fluorescent Protein (ECFP) were chosen for localization analyses and for in vivo protein-protein interaction studies by FRET, HA and cMyc were selected as epitope tags for immunoprecipitation, and GST and STREP (streptavidin) tags were chosen for affinity purification assays. Each tag was cloned in both the 5' and 3' configuration resulting in 12 different Entry cassettes (Figure 1). All the tags, except STREP, were PCR amplified from pre-existing vectors as *Sal *I-*Bam*H I fragments for N-terminal tagging and as *Not *I-*Xho *I fragments for C-terminal tagging. These latter ones have been amplified in order to contain a stop-codon immediately after the tag so that any gene of interest can be cloned in all the 12 different Entry plasmids as *Bam*H I – *Not *I fragments without a stop codon. The STREP tag was generated instead by annealing two pairs of oligonucleotides, one for N-terminal and one for C-terminal tagging following the same restriction enzyme strategy described above. The oligonucleotides used for generating epitope tags are listed in Table 1.

**Table 1:** Oligonucleotides used to generate epitope tags. Restriction sites are in bold and linker sequences are underlined.

**Oligo name**	**Sequence 5'-3'**
nSTREPfw	**TCGAC**ATGTGGAGCCATCCGCAGTTCGAAAAAGGCGGCAGCGGC**G**
nSTREPrev	**GATCC**GCCGCTGCCGCCTTTTTCGAACTGCGGATGGCTCCACAT**G**
nHAfw	GAA**GTCGAC**ATGTCGCGATACCCCTAC
nHArev	GAA**GGATCC**TCCACTGCTAGCGGCGTAG
nMycfw	GA**GTCGAC**GGTATCGATTTAAAGC
nMycrev	GAA**GGATCC**CGGGCTTCCGGAATTCAAGTCCTCTTC
nGSTfw	GAA**GTCGAC**ATGTCCCCTATACTAGGTTATTG
nGSTrev	ACG**GGATCC**ACGCGGAACCAGATC
nYFPfw	GG**GTCGAC**ATGGTGAGCAAGGGCG
nYFPrev	CC**GGATCC**AGGCTTGTACAGCTCGTCCATG
cSTREPfw	**GGCCGC**AAGCGGCGGATGGAGCCATCCGCAGTTCGAAAAATAG**C**
cSTREPrev	**TCGAG**CTATTTTTCGAACTGCGGATGGCTCCATCCGCCGCTT**GC**
cHAfw	A**GCGGCCGC**AAGCGGAGGCCTGTCGCGATAC
cHArev	GAA**CTCGAG**AGTACTGCTCTAGGCTTAGTCGGGCAC
cMycfw	GAA**GCGGCCGC**ACAAGCTATGGAGCAAAAGC
cMycrev	GAA**CTCGAG**TCAGGAATTCAAGTCCTC
cGSTfw	A**GCGGCCGC**AAGCGGAGGCATGTCCCCTATACTAGG
cGSTrev	GAA**CTCGAG**CTAATCCGATTTTGGAGGATGG
cYFPfw	GG**GCGGCCGC**ACCTATGGTGAGCAAGGGCG
cYFPrev	CC**CTCGAG**TTACTTGTACAGCTCGTCCATG

**Figure 2:**
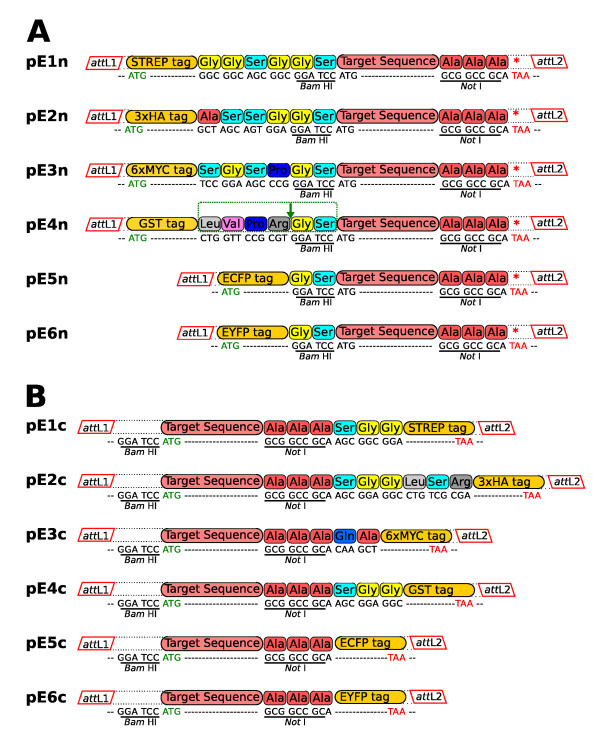
**Modified pENTR vectors**. Epitope tags were introduced in the pENT-STOP vector between the *att*L1 and *att*L2 recombination sites to generate modified pENTR vectors both for N-terminal (A) and C-terminal (B) tagging (pE1n-pE6n and pE1c-pE6c respectively). The amino-acid composition of the short linkers added between the tag and the protein and their reading frame with respect to the target sequence is shown for all the constructs. The *Bam*H I and N*ot *I sites are underlined. A thrombin cleavage site is present in pE4n for protein purification purposes.

The main aim of this modified Gateway cloning strategy is to have the desired open reading frame (ORF) encoding the tagged protein of interest within the Entry vector to avoid the presence of additional, often highly charged, sequences after the recombination event that could interfere with protein function. However, in some cases the presence of a flexible linker between the tag and the protein is very useful to permit appropriate folding of the fusion protein. To this end the primers used to amplify the tag sequences have been designed to create a short linker between the gene and the tag and, for purification purposes, a thrombin cleavage site Leu-Val-Pro-Arg-Gly-Ser has been added to the N-terminal GST tag (Figure 2, Table 1).

In order to test this system a number of tomato genes currently under investigation in our laboratory, such as encoding the core histones H2A [9] and H2B [10], ubiquitin [11], DET1 (De-ETiolated1) [12] and DDB1 (UV-Damaged DNA Binding protein 1) [13] were cloned by REaL into the newly created Entry vectors (Table 2).

**Table 2:** Tests of functionality of the newly generated constructs.

**REal – derived pENTR constructs**	**SSR – derived pDEST construct**	**Expression in *N. benthamiana***	**Expression in *Arabidopsis***
pE2n-H2A	pH2n-H2A	Yes	Yes
pE2c-H2A	pH2c-H2A	Yes	Yes
pE2n-H2B	pH2n-H2B	Yes	Yes
pE2c-H2B	pH2c-H2B	Yes	Yes
pE1c-H2B	pB1c-H2B	NT	Yes
pE2n-UBI	pH-2n-UBI	Yes	Yes
pE6n-UBI	pB6n-UBI	NT	Yes
pE5n-UBI	pH5n-UBI	NT	Yes
pE1n-UBI	pB1n-UBI	NT	Yes
pE3n-DET1	pK3n-DET1	Yes	NT
pE6n-DET1	pB6n-DET1	NT	No
pE6n-DET1	pM6n-DET1	NT	Yes
pE1n-DET1	pB1n-DET1	Yes	No
pE2n-DDB1	pH2n-DDB1	Yes	NT
pE3n-DDB1	pK3n-DDB1	Yes	NT
pE2c-DDB1	pH2c-DDB1	Yes	NT
pE3c-DDB1	pK3c-DDB1	Yes	NT

Table shows constructs made and their functionality by expression analysis in *N. benthamiana *and in transgenic *Arabidopsis *plants. Protein extracts from transiently transformed tobacco leaves or from Arabidopsis homozygous lines were analysed by western blotting using the appropriate antibodies. Yes, expressed. No, not expressed. NT, not tested. pH, pH2GW7. pK, pK2GW7. pB, pB2GW7. pM, pMDC7.

**Figure 3:**
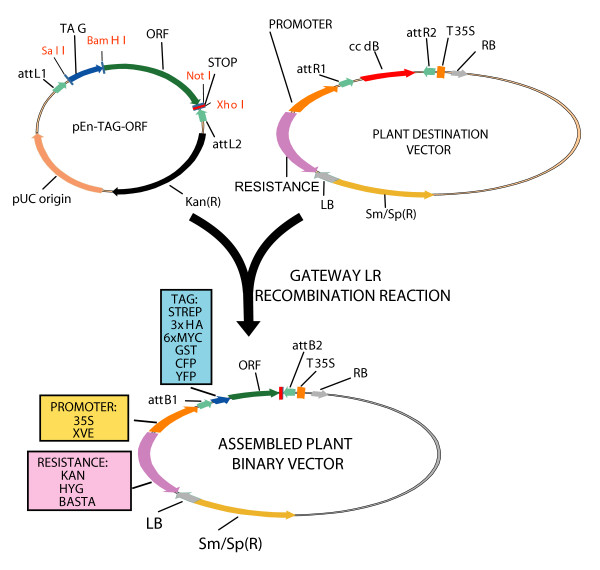
**Recombination event in plant binary Destination vectors**. Schematic drawing of the recombination between the newly generated pENT-TAG-GENE vectors and a Destination vector. The recombination events between the *att*L and *att*R sites that generate *att*B sites occur outside the ORF encoding the tagged protein of interest so no additional sequences are added. A variety of assembled plant binary vectors can be generated with different promoters and resistance markers.

The modified Entry vectors containing the tagged ORFs of interest were then recombined by SSR into binary Gateway overexpression vectors pB2GW7, pH2GW7 or pK2GW7 from the Laboratory of Plant Systems Biology (PSB; Ghent University, Belgium) (Figure 3). These vectors have a 35S Cauliflower Mosaic Virus (CaMV) promoter/terminator and either a hygromycin-B resistance cassette (pH2GW7), a kanamycin resistance cassette (pK2GW7) or a Basta resistance cassette (pB2GW7). [3]. For inducible gene expression the Gateway Destination vector pMDC7 (Ueli Grossniklaus; Zurich, Switzerland) was used. This vector contains the XVE inducible promoter for 17-β-estradiol inducible expression in plants and the hygromycin B resistance gene [4,14]. We chose to use these binary Destination vectors over others due to the flexibility they offer with respect to selectable markers and promoters. A further advantage is that they have a spectinomycin/streptomycin bacterial selectable marker whereas the Entry vectors contain a kanamycin resistance cassette, thus allowing easy selection of the correct recombined product after the Gateway recombination reaction. However, pCAMBIA-derived Gateway overexpression vectors [4,5] could also be used provided that the Entry vector is linearised prior to recombination (this step is necessary because pCAMBIA has a kanamycin bacterial selectable marker). Finally, these modified Entry vectors can be recombined into common overexpression vectors in experimental systems other than plants such as bacteria (pDEST14), mammalian cells (pDEST32), and insect cells (pDEST8) [2].

**Figure 4:**
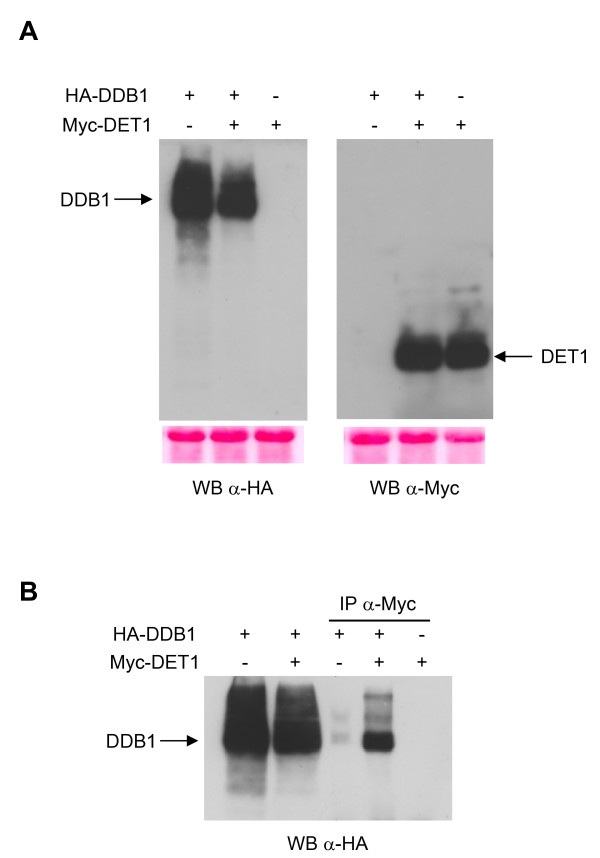
**Transient expression in *Nicotiana benthamiana *tobacco leaves**. A, Protein extracts (20 μg) from tobacco leaves transiently transformed with pK3nDET1 and pH2nDDB1 either alone or in combination were separated on 10% SDS-PAGE and analysed by western blotting using antibodies against HA and c-Myc as indicated. Ponceau staining is shown as loading control below each panel. B, Immunoprecipitation analysis of protein extracts (200 μg) from leaves transformed with the indicated tagged proteins with c-Myc antibody. Total extracts (30 μg) and immunoprecipitated samples were resolved on 10% SDS-PAGE and probed with α-HA antibody.

Table 2 summarizes the different Destination vectors that were generated by SSR and which were introduced into *A. tumefaciens *for infiltration of *N. benthamiana *leaves in order to verify their expression. Figure 4A shows an example of transient expression analysis in which pE3n(6xMYC)DET1 and pE2n(3xHA)DDB1 were recombined in pK2GW7 and pH2GW7, respectively. The recombined pDEST vectors, pK3nDET1 and pH2nDDB1, were agro-infiltrated alone or in combination in tobacco leaves. Total proteins extracted from infiltrated leaves were resolved on 10% SDS-PAGE and analysed by western blot using antibodies against the cMyc and HA epitopes. In order to confirm that the expressed proteins were functional we tested the interaction between DET1 and DDB1, which are known to interact [15], by co-immunoprecipitation. Total protein extracts were immunoprecipitated with an antibody against cMyc to pull down DET1, and samples were then analysed by western blotting with an antibody against HA to verify the presence of DDB1. The results show that DDB1 efficiently co-immunoprecipitated with DET1, demonstrating that the two proteins were indeed functional by this criteria (Figure 4B).

**Figure 5:**
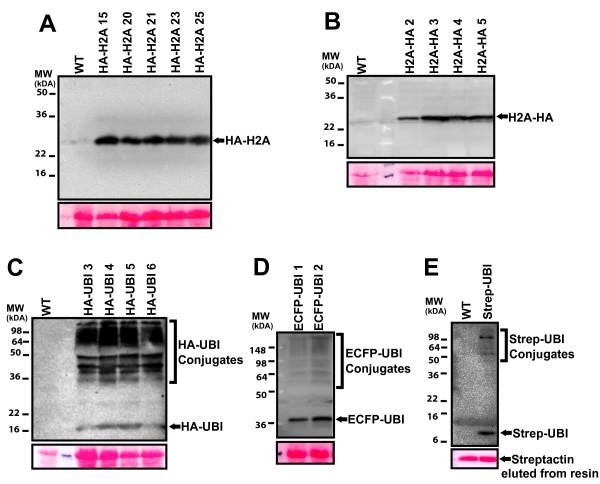
**Expression analysis of transformed Arabidopsis lines**. Protein extracts (20 μg) from wild type (WT) and different independent Arabidopsis transgenic lines transformed with pH2n-H2A (A), pH2c-H2A (B), pH2n-UBI (C), pE5n-UBI (D) were separated on 15% SDS-PAGE and analysed by western blotting using antibodies against HA (A-C) or GFP (D). Ponceau staining is shown as loading control below each panel. E, Protein extracts from wild type (WT) and an Arabidopsis transgenic line expressing Strep-Ubiquitin were used for affinity purification on a Streptactin resin. Bound proteins were resolved on 15% SDS-PAGE and analysed by western blotting using an antibody against the STREP epitope. Ponceau staining at the bottom shows the presence of streptactin in both samples.

A range of recombined pDEST vectors were also used to transform Arabidopsis plants using the floral dip method [1]. Figure 5 shows examples of analysis of the generated Arabidopsis lines. Tomato histone H2A was cloned in both pE2n(3xHA) and pE2c(3xHA) and then recombined in pH2GW7 to generate pH2n-H2A and pH2c-H2A, respectively, that were then used to transform Arabidopsis plants. Several independent lines were selected on hygromycin and protein extracts from homozygous lines were analysed by western blotting with an antibody against HA to reveal the presence of the protein of interest (Figure 5A,B). Another series of transgenic lines expressing ubiquitin was also analysed. In this case tomato ubiquitin was cloned in pE2n(3xHA), pE5n(ECFP) and pE1n(STREP) and recombined in pH2GW7 and pB2GW7 destination vectors. The resulting plasmids pH2n-UBI, pH5n-UBI and pB1n-UBI were transformed in Arabidopsis plants and homozygous lines were selected on the appropriate antibiotic. Protein extracts from homozygous lines were analysed by western blotting with antibodies recognizing the HA tag (Figure 5C) or the ECFP tag (Figure 5D). With both antibodies we were able to visualize both ubiquitin monomers and higher molecular weight conjugates likely representing ubiquitinated cellular proteins. In order to test the functionality of the Strep-tagged construct a homozygous Arabidopsis line expressing Strep-Ubiquitin was used for affinity purification (Figure 5E). Protein extracts from WT and a transformed line were incubated with a Streptactin resin and bound proteins were then analysed by western blotting using an antibody against the Strep epitope tag. The results presented in Figure 5E show that the tagged protein was efficiently retained on the Streptactin resin demonstrating the potential use of this vector for affinity purification in plants.

Table 2 summarizes the extent to which the constructs described herein have been tested and shown to be functional, confirming the utility of the system we describe.

## Discussion

The hybrid cloning system reported here, based both on REaL and SSR approaches, enables the straightforward generation of protein-tag fusions of a protein of interest with a number of N- or C-terminal tags. The REaL-generated cassettes can then be recombined by SSR into vectors containing the appropriate promoter and resistance combinations suited to the particular experimental situation. The major advantage of our system with respect to conventional Gateway-based cloning systems for plant genes [5,6], is that the *att*-derived linker sequences, which may adversely affect protein functionality, are avoided in the resultant protein fusions. A further advantage is that a sequence of interest can be fused to all of the six tags, either as N- or C-terminal fusions, using the same strategy because all the vectors use the same reading frame and because stop codons are present in all the modified pENTR vectors. Disadvantages with respect to conventional plant-based Gateway cloning are that all the pENTR vectors are designed for cloning with only *Bam*H I and *Not *I (additional restriction sites can be added, although this will obviously modify the linker sequence between the protein and the tag), and that a REaL cloning step is also required. Consequently it should be viewed as being complementary rather than as a substitute for the more conventional Gateway-based systems because it expands the choice of tags and higher orders of combinations, and permits more control over the linker sequence lying between a protein of interest and an epitope tag.

We have demonstrated the utility of this vector system for both stable and transient *Agrobacterium*-mediated plant transformation, although the flexibility of the system allows a number of other possibilities to be explored. For example, these cassettes could also be used for direct (biolistic bombardment or poly-ethylene-glycol (PEG)-mediated) transformation of plant cells or protoplasts either using the binary vectors themselves or by recombining them into Gateway minimal overexpression vectors optimized for direct transient transformation, available from the Dept of Plant Systems Biology at the University of Ghent (Belgium) [16].

Alternatively, although strong viral promoters such as the 35S promoter have been widely and successfully used for expression of transgenes in plants, such high levels of expression can lead to unwanted side effects and lead to misleading results. In many cases it is therefore more desirable to express transgenes from their endogenous promoters. To this end, an improved "MultiSite Gateway" system has recently been developed [17] and incorporated into plant binary vectors [18]. As its name suggests, the MultiSite Gateway system makes use of multiple unique *att *sequences (*att*1–4) which allow two or three separate fragments to be simultaneously transferred to a Destination vector via the Gateway recombination reaction. In addition, the desired promoter fragment can be cloned into a separate Entry vector containing the *att*L4 and *att*R1 sites or may already be available as part of the SAP project (Systematic Analysis of Promoters) [19]. The promoter fragment can then be recombined together with the existing tag-protein Entry cassette into a Gateway multi-site binary Destination vector (containing a transcriptional terminator and the *att*R2 and *att*R4 sites) to yield the protein fused to the desired N- or C-terminal tag under the control of its own promoter. Although not yet tested by us, these Destination vectors add a further level of versatility that can be exploited in combination with the Entry vectors described here.

While expression of recombinant proteins *in planta *has many advantages, it is currently not suitable for generating the large amounts of recombinant protein needed for *in vitro *investigations or antibody generation. To address this difficulty our tag-protein fusion cassettes can be recombined into vectors for in vitro expression or for overexpression in bacterial (pDEST14), yeast, insect (pDEST8) or mammalian cell systems (pDEST32). We have not directly tested this, but it should nonetheless be feasible to try several expression systems in parallel to determine which system is most suited for expression of the protein of interest because the only requirement is a Gateway Destination vector adapted to a particular expression system.

## Conclusion

The modified pENTR vectors described here are highly complementary to the more conventional SSR-based Gateway systems in that they expand the range of possibilities for experimentation, e.g., when combined with different promoter and antibiotic resistance cassettes offered by Gateway Destination vectors. They also overcome some of the disadvantages of previously described Gateway systems, but because the hybrid system we report is somewhat more labor intensive than classical SSR cloning, we consider it best suited to low-medium throughput applications. For example, we believe it can be of great utility for studying protein complexes because it can allow studies of protein:protein interactions and subcellular localisation in a variety of systems and combinations, without the presence of bulky *att*-derived linker sequences that may interfere with protein activity.

## Methods

### Construction of modifed Gateway Entry cassettes

pENT-Stop was created by annealing the two oligonucleotides ENT-StopFw 5'-GATCCGGTACAGATTTCGGACATGCGGCCGCATAAGTAGCTGAC-3' and ENT-StopRev 5'-TCGAGTCAGCTACTTATGCGGCCGCATGTCCGAAATCTGTACCG-3' and ligating the resultant fragment into *Bam*H I – *Xho *I digested pENTR-1A vector (Invitrogen; Carlsbad, CA). This introduces a *Not *I site followed by a stop codon and removes the *ccdB *toxicity gene. This plasmid has been used for cloning the different epitope tags. The N-terminal STREP tag was created by annealing two oligonucleotides (see Table I) and ligating the resultant *Sal *I – *Bam*H I fragment into the pENT-Stop. The other N-terminal tags were PCR amplified with primers that introduce a 5' *Sal *I site and a 3' *Bam*H I site.

The C-terminal STREP tag was created by annealing two oligonucleotides (see Table I) and ligating the resultant *Not *I – *Xho *I fragment into the pENT-Stop. The other C-terminal tags were PCR amplified with primers that introduce a 5' *Not *I site and a 3' *Xho *I site. Oligonucleotides used for PCR amplifications are listed in Table I. The PCR products were ligated into the pCR2.1 vector using the Topo-TA cloning system (Invitrogen; Carlsbad, CA) and sequenced. The tags were then excised from pCR2.1 using the appropriate restriction enzymes and cloned into the pENT-Stop vector. Genes of interest were amplified by PCR from pre-existing plasmids with gene-specific primers introducing a 5' *Bam*H I site and a 3' *Not *I site.

Tag-ORF fusions were transferred from the newly generated Entry vectors to a plant binary Destination vector with the desired promoter and selective marker using the Gateway LR reaction and the Recombinase enzymes (Invitrogen; Carlsbad, CA). The identity of recombined vectors was verified by PCR screening and restriction enzyme analysis.

### Plant material and *Agrobacterium*-mediated transformation

*Arabidopsis thaliana *(ecotype Columbia) and *Nicotiana benthamiana *were grown in soil in a growth room at 22°C under long day conditions (16/8 hrs light dark cycle). Arabidopsis plants were transformed by the floral dip method [1]. Transgenic lines were selected with the appropriate resistance agent (50 μg/ml kanamycin, 10 μg/ml hygromycin B or 10 μg/ml Basta) and tested for expression. *Nicotiana benthamiana *leaves were infiltrated with *A. tumefaciens *strain GV3101 harbouring recombined pDEST vectors. GV3101 cells were grown in 10 ml LB broth with antibiotic overnight at 28°C and then pelleted at 2,500 *g *for 10 min. Cells were then washed in 10 ml of infiltration medium (50 mM MES pH 5.7, 0.5% glucose, 2 mM NaH_2_PO_4 _and 200 μM acetosyringone), centrifuged again at 2,500 *g *for 5 min and resuspended in infiltration medium at an OD_600 _of 0.3. The *Agrobacterium *solution was infiltrated in the abaxial surface of the leaf using a 1 ml syringe without needle. After infiltration plants were placed under normal light conditions and leaves were collected after 36–48 hours for further analysis.

### Protein extraction, western blotting and immunoprecipitation analysis

Rosette leaves (approximately 2 cm^2 ^in total) were collected from individual 3 week old homozygous lines, ground in liquid nitrogen to fine powder and then resuspended in 50 μl 1 × Laemmli buffer (62.5 mM Tris-HCl (pH 6.8), 2% (w/v) SDS, 10% (v/v) glycerol, 1% (v/v) β-mercaptoethanol, 0.0005% (w/v) bromophenol blue).

Transformed tobacco leaves were ground in liquid nitrogen and then resuspended in Lysis buffer (50 mM Tris pH 7.8, 10 mM EDTA, 300 mM sucrose, 100 mM NaCl, 1 mM DTT, protease inhibitor cocktail (Roche)). Samples were then cleaned by successive centrifugations at 1,000, 2,000 and 10,000 *g*. For western blotting analysis samples were separated on SDS-PAGE and proteins were transferred onto PVDF membranes by semidry electroblotting. For detection of proteins the antibodies used were: mouse anti-HA (Covance) at 1:3,000 dilution, mouse anti-c-Myc (Upstate) at 1:2,000 dilution, rabbit anti-GFP (Abcam) at 1:10,000 dilution and mouse anti-Strep (IBA, Göttingen, Germany) at 1:5,000 dilution. As secondary antibodies anti-mouse and anti-rabbit (Promega) were used both at 1:10,000 dilution. Blots were developed using an ECL kit (GE Healthcare; Little Chalfont, UK).

For immunoprecipitation analysis 300 μg of total proteins were incubated overnight at 4°C with rabbit anti-Myc antibody (Abcam) at 1:400 dilution in lysis buffer and then incubated with protein-A Sepharose (Amersham) for 2 hours at 4°C. The immunoprecipitates were washed three times with lysis buffer, resolved on 10% polyacrylamide gel and subjected to western blotting analysis as described above.

For Strep-tagged protein purification 200 ng of plant tissue from 3-week old seedlings were ground to fine powder in liquid nitrogen and resuspended in 0.5 ml Ex-strep buffer (100 mM Tris-HCl pH 8.0, 5 mM EGTA, 5 mM EDTA, 150 mM NaCl, 10 mM DTT, 0.5 mM AEBSF, 0.5% (v/v) Triton-X100, 100 μg/ml avidin, protease inhibitor cocktail (Roche)). The lysate was sonicated 3 times for 10 seconds (30% output on a Branson Sonifier 250 (Branson Ultrasonics; Danbury, CT)) on ice with a 50 seconds pause between pulses. The lysate was centrifuged at 21,000 *g *for 10 min at 4°C and the supernatant was then incubated with 30 μl of Streptactin Sepharose beads (IBA; Göttingen, Germany) for 1 hour at 4°C with gentle rotation. The beads were washed 4 times with 0.75 ml W-strep buffer (50 mM Tris-HCl pH 8.0, 2.5 mM EDTA, 150 mM NaCl, 2 mM DTT and 0.05% (v/v) Triton-X100). Bound proteins were then eluted in 60 μl of 1× Laemmli buffer at 95°C for 10 min and analysed by western blotting as described above.

### Plasmids availability

The twelve pENTR plasmids described herein will be made available for noncommercial research purposes at Addgene [20]. Accessions numbers are as follows: pE1n, (GenBank:EU334816). pE2n, (GenBank:EU334817). pE3n, (GenBank:EU334818). pE4n, (GenBank:EU334819). pE5n, (GenBank:EU334820). pE6n, (GenBank:EU334821). pE1c, (GenBank:EU334822). pE2c, (GenBank:EU334823). pE3c, (GenBank:EU334824). pE4c, (GenBank:EU334825). pE5c, (GenBank:EU334826). pE6c, (GenBank:EU334827).

## Competing interests

The author(s) declare that they have no competing interests.

## Authors' contributions

MJD: experimental design, vector construction, Arabidopsis data and manuscript preparation. CB: experimental design and manuscript preparation. GB: experimental design, *Nicotiana benthamiana *data and manuscript preparation. All authors read and approved the final manuscript.
